# Embolization of a wide-necked aneurysm of the renal artery ostium using an Amplatzer vascular plug 2: a case report

**DOI:** 10.1186/s42155-025-00607-1

**Published:** 2026-01-07

**Authors:** Mario Ghosn, Haytham Derbel, Youssef Zaarour, Félix Wei, Vania Tacher, Hicham Kobeiter

**Affiliations:** 1 Department of Radiology, Henri Mondor Hospital, Assistance Publique — Hôpitaux de Paris (AP-HP), Créteil, France; 2 Université Paris-Est Créteil (UPEC), Créteil, France

**Keywords:** Renal aneurysm, Abdominal aorta, Vascular plug, Endovascular, Embolization

## Abstract

**Background:**

This report describes an embolization technique using a steerable sheath and an Amplatzer vascular plug 2 (AVP 2) to treat a wide-necked aneurysm located at the ostium of the renal artery. Given this particular location, directly attached to the aortic wall, similar to a saccular aneurysm of the abdominal aorta, stent placement or embolization using coils or liquid agents was not feasible.

**Case presentation:**

A 45-year-old patient with a long medical history including heterozygous SC sickle cell disease, systemic lupus erythematosus, and a left iliac fossa kidney transplant presented with a partially thrombosed right renal artery aneurysm of 45-mm diameter. The aneurysm was located at the ostium of the renal artery that was occluded downstream. The aneurysm was directly attached to the aortic wall with a wide neck measured at 8 mm. Use of coils or liquid agents was not possible because of a very high risk of extra-target embolization. Lack of a patent right renal artery downstream precluded placement of a covered stent. Following multidisciplinary discussion, and due to the patient’s high risk for aortic abdominal surgery, endovascular management with embolization was decided. Embolization was performed under local anesthesia, using fluoroscopic guidance and a cone-beam computed tomography three-dimensional road map. Following common right femoral artery access, a 7F steerable sheath was used to catheterize the aneurysm. An AVP 2 was then passed through the sheath in the aneurysm. Particular attention was paid to deploying the last disc of the AVP 2 in the aortic lumen to ensure closure of the aneurysm neck. Final aortic angiogram confirmed exclusion of the aneurysm. There were no intraoperative or postoperative complications. At computed tomography performed 7 months later, the AVP 2 remained in position, and the aneurysm was excluded and partially decreased in size.

**Conclusions:**

In an anatomical presentation that was not a candidate for stent placement or classic embolization techniques, deployment of an AVP 2 using a steerable sheath successfully excluded the aneurysm. This procedure, performed under local anesthesia, obviated the need for abdominal aortic surgical repair or for an aortic stent graft.

## Background

Renal artery aneurysms (RAA) rupture can lead to a patient’s death or loss of a kidney. According to the Cardiovascular and Interventional Radiological Society of Europe, RAA must be treated if it has a size > 2 cm [1]. Endovascular treatment has a high technical success rate [2–4], with different techniques reported, including coiling [5, 6], use of liquid embolic agents [2], and stent grafts [7].

This study describes an embolization technique using an Amplatzer vascular plug (AVP) 2 (Abbott Cardiovascular, Chicago, IL, USA) to treat a wide-necked aneurysm of the renal artery ostium that presented as a saccular aneurysm of the abdominal aorta. As this anatomical presentation was not a candidate for the embolization techniques mentioned above, this approach obviated the need for abdominal aortic surgical repair or for an aortic stent graft.

## Case presentation

**Fig. 1:**
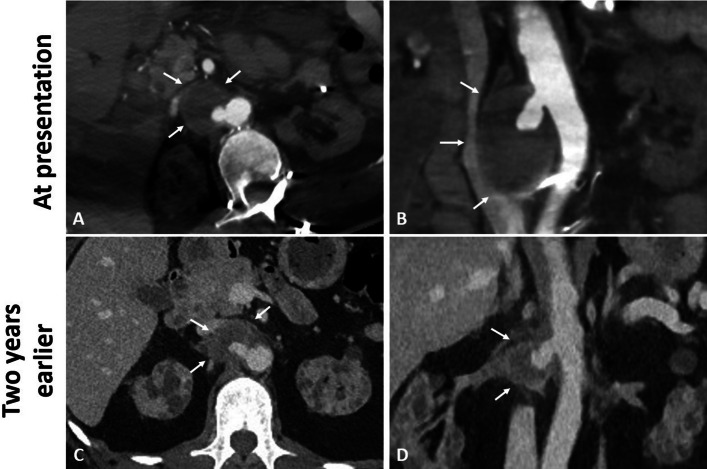
Aneurysm of the ostium of the right renal artery. Axial (**A**) and coronal (**B**) CT slices showing a 45 mm partially thrombosed aneurysm (arrows) located at the ostium of the right renal artery. The right renal artery was occluded downstream of the aneurysm. The aneurysm had a neck measuring 8 mm. Two years before, the aneurysm (arrows) measured 23 mm, as shown on axial (**C**) and coronal CT slices (**D**)

A 45-year-old patient who had undergone a left iliac fossa kidney transplant 3 days ago underwent a computed tomography (CT) scan that showed a partially thrombosed right RAA (Fig. 1). The RAA had a diameter of 45 mm, with a canalized portion of 16 mm, compared to a diameter of 23 mm on the CT scan 2 years ago. The aneurysm was located at the ostium of the renal artery that was occluded downstream, with a wide neck measured at 8 mm (Fig. 1). The right kidney was atrophic and non-functional.

The patient had a long medical history including heterozygous SC sickle cell disease, systemic lupus erythematosus, chronic renal failure undergoing dialysis, arterial hypertension, diabetes, several ischemic strokes, superior vena cava syndrome requiring curative anticoagulation, and a seronegative antiphospholipid syndrome.

**Fig. 2:**
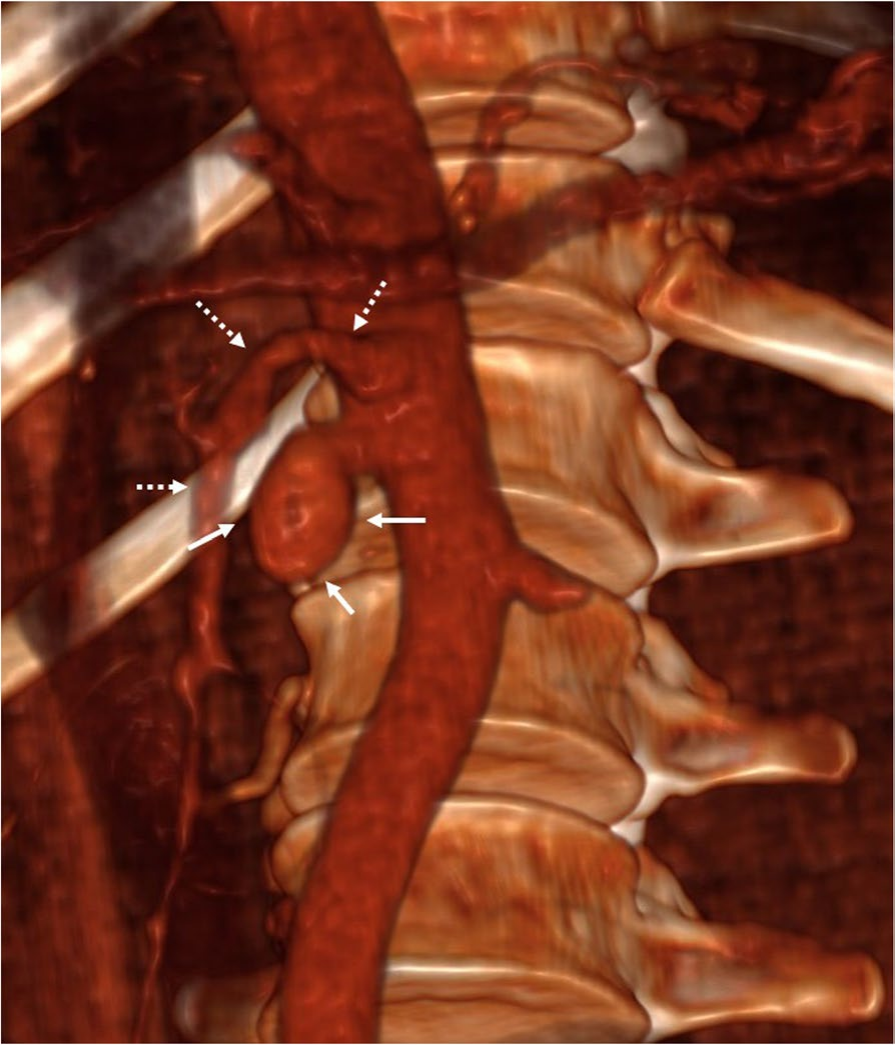
Three-dimensional computed tomography reconstruction of the aneurysm at the ostium of the right renal artery. Three-dimensional CT reconstruction illustrating the aneurysm of the right renal artery (white arrows) and its spatial relationship with the superior mesenteric artery (dashed white arrows)

Due to its anatomical presentation, similar to a saccular aneurysm of the abdominal aorta [8], an aortic stent graft or an open surgical repair should have been the treatment of choice (Fig. 2). However, given the anesthetic and operative risks, a multidisciplinary team with anesthesiologists, surgeons, and interventional radiologists opted for an endovascular embolization.

**Fig. 3:**
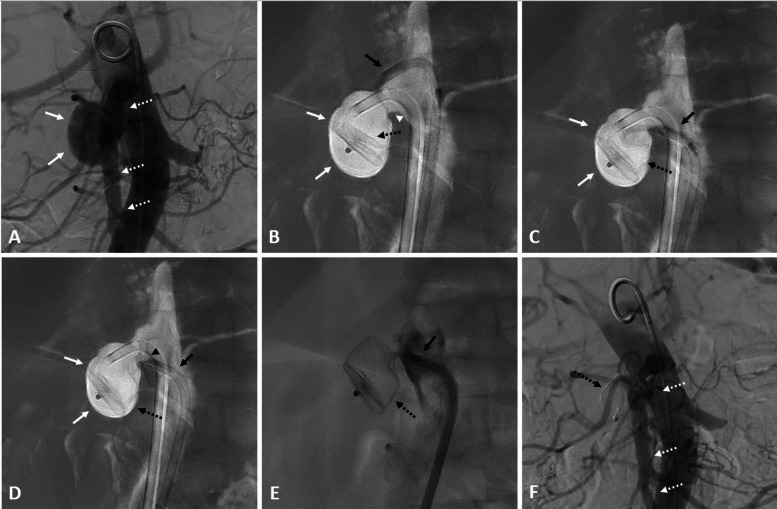
Endovascular embolization of the aneurysm using an Amplatzer vascular plug 2. **A** Aortic angiogram showing the right renal artery aneurysm (white arrows) and its relation to the ostium of the superior mesenteric artery (white dashed arrows). **B** A two-dimensional road map showing the opacified aneurysm (white arrows), its neck (white arrowhead), and its relation to the aortic wall was used throughout the procedure. The aneurysm was catheterized using only the steerable sheath (black arrow). Once the steerable sheath extremity was stabilized in the bottom of the aneurysm, the AVP 2 (black dashed arrow) was deployed inside the aneurysm, starting from its distal side. **C** Once the AVP 2 (black dashed arrow) was almost entirely deployed in the aneurysm (white arrows), the steerable sheath (black arrow) was pulled back. **D** Steerable sheath (black arrow) was pulled back to deploy the last disc (black arrowhead) of the AVP 2 (black dashed arrow) outside the aneurysm, i.e., in the aortic lumen, to ensure closure of the aneurysm (white arrows) neck. **E** Injection through the sheath (black arrow) confirmed the exclusion of the aneurysm by the AVP 2 (black dashed arrow). **F** Final aortic angiogram confirmed exclusion of the aneurysm by the AVP 2 (black dashed arrow) and patency of the adjacent SMA (white dashed arrows)

The technique was inspired by percutaneous patent foramen ovale closure procedures [9]. Under local anesthesia and using ultrasound guidance, the common right femoral artery was punctured, and a 7F steerable sheath (Aptus Tour Guide, Medtronic, Dublin, Ireland) of 55-cm length was introduced. A 5Fr pigtail catheter was then placed above the superior mesenteric artery (SMA), and an aortic angiogram was obtained using 20 mL of Visipaque 320 mg I/mL (iodixanol, GE HealthCare, Wauwatosa, WI, USA) injected at a rate of 5 mL/s (Fig. 3). Aortic angiogram confirmed the presence of the partially thrombosed RAA aneurysm (Fig. 3).

An unenhanced cone-beam computed tomography (CBCT) of the abdomen was then obtained. Using EmboGuide software (EmboGuide, Philips Healthcare, Best, The Netherlands), the interventional radiologist marked the neck of the aneurysm on available three-dimensional images. The landmarks of the aneurysm neck were then overlaid on live fluoroscopy, which allowed the operator to choose the fluoroscopic incidence that best showed the neck of the aneurysm and its position in relation to the aortic wall. Using this working incidence, a two-dimensional road map showing the filled aneurysm was then obtained and used throughout the rest of the procedure (Fig. 3).

The 5Fr pigtail catheter was removed, and the right RAA was catheterized using only the steerable sheath, without the need for a guidewire. Indeed, the sheath extremity was orientated by the operator in order to be stabilized in the bottom of the aneurysm (Fig. 3). An AVP 2 of 20-mm diameter and 16-mm length was then passed through the sheath in the aneurysm. The diameter of the AVP was chosen on the basis of two factors: it had to have a diameter greater than that of the circulating portion of the aneurysm, but also not be too long in order to exit into the aortic lumen without overflowing into adjacent vessels, notably the SMA in this patient. While most of the AVP was deployed inside the aneurysm, particular attention was paid to deploying the last disc outside the aneurysm, i.e., in the aortic lumen, to ensure closure of the aneurysm neck.

After deployment, an aortic angiogram was again performed and confirmed exclusion of the aneurysm and patency of the adjacent SMA (Fig. 3). An unenhanced cone-beam computed tomography ensured correct positioning of the AVP 2 and no obstruction of the ostium of the SMA. Material was then removed, and the femoral access was closed using an Angio-Seal 8F closure device (Terumo, Somerset, NJ, USA).

**Fig. 4:**
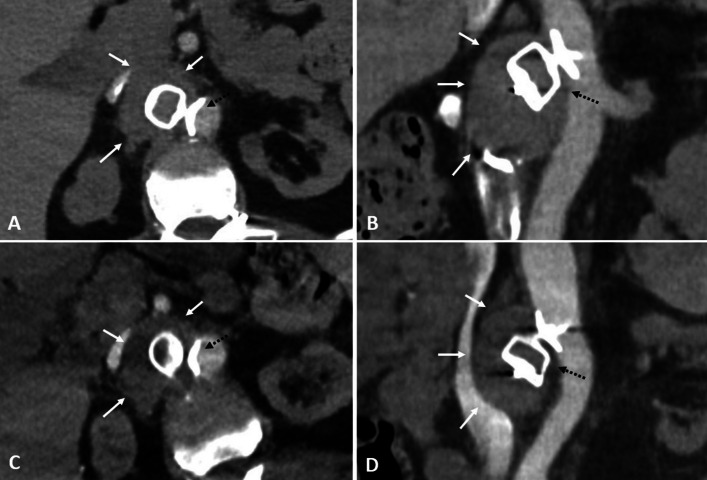
Follow-up imaging confirming complete exclusion of the aneurysm. Follow-up CT performed 4 days after the embolization confirmed the exclusion of the right renal artery aneurysm (white arrows) and the correct positioning of the AVP 2 (black dashed arrow), here shown on axial (**A**) and coronal (**B**) slices. CT performed 7 months later showed that the AVP 2 (black dashed arrow) remained in position, and that the aneurysm (white arrows) partially decreased in size and remained excluded, as illustrated on axial (**C**) and coronal (**D**) slices

There were no intraoperative or postoperative complications. Due to several coagulation-related diseases, the patient was under curative anticoagulation therapy that could not be stopped and was thus continued following embolization. The first follow-up CT performed 4 days after the procedure confirmed the complete exclusion of the RAA (Fig. 4). AVP 2 remained correctly positioned, identical to per-procedural cone-beam computed tomography (CBCT), with no migration. The AVP 2 overflowed slightly onto the lower part of the ostium of the SMA, with a nonsignificant stenosis < 5%.

Another CT performed 7 months later also showed that the AVP 2 remained in position, and that the aneurysm remained excluded (Fig. 4). The size of the aneurysm partially decreased, with a maximum diameter of 42 mm. Over the entire 8 months of follow-up, the patient did not show any sign of mesenteric ischemia.

## Discussion

Using an AVP 2 and a steerable sheath, and under local anesthesia, embolization was successful and allowed the patient to avoid aortic repair and its potential morbidity. In this patient, the RAA was located at the ostium of the renal artery, which was occluded downstream of the aneurysm, and the concerned kidney was atrophic. The presentation was thus similar to a saccular aneurysm of the abdominal aorta [8]. The Society for Vascular Surgery practice guidelines recommend endovascular aortic repair or open repair as the two treatment modalities for saccular aortic aneurysms [10].

Multiple endovascular techniques have been reported to treat renal artery aneurysms [4], but none was suitable for this case. Embolization using coils was estimated to not be feasible because the neck of the aneurysm was large with a high risk of coil migration. The use of liquid embolic agents carried the same risk of nontarget embolization. A stent graft could not be deployed because the renal artery was occluded downstream of the aneurysm, so there was no landing zone.

Several treatment options for the RAA were evaluated by the multidisciplinary team. A “cage and coil” approach or the use of a short uncovered aortic stent with transstent coil embolization of the aneurysm sac could have been performed under local anesthesia. However, these strategies were not feasible due to the wide aneurysm neck and the high risk of coil migration [11]. In addition, the use of a short uncovered aortic stent would have increased morbidity, as it requires a larger introducer sheath with a risk of covering the ostium of the SMA [11, 12]. Placement of a conventional aortic endograft was also deemed unfeasible due to the insufficient neck near the SMA and the need for a large sheath, which posed a risk of arterial occlusion of the kidney transplant. Other potential endovascular options included the use of a physician-modified aortic endograft with SMA fenestration or a custom-made stent graft. However, because of the time required for CMD production and, more importantly, the increased complexity associated with prolonged mesenteric and renal ischemia, these procedures were considered by the MDT as secondary options, to be pursued only in the event of AVP 2 failure. Of note, the RAA diameter measured 2.3 cm prior to the kidney transplant. Ideally, this issue should have been addressed earlier, and an open repair could have been performed concurrently with the transplant.

Use of vascular plugs, as previously described in renal arteries [13], was deemed not feasible in this anatomical location using a standard sheath or catheter. Indeed, in the absence of a downstream artery, the used sheath must be steerable to allow catheterization of the aneurysm ostium and also provide sufficient stability to allow AVP 2 deployment. Several technical points must be considered with this technique. The AVP 2 diameter must be larger than the aneurysm neck to ensure complete occlusion. Care must be taken when choosing the length of the AVP 2: it should not extend too far into the abdominal aorta to avoid covering the ostia of other visceral arteries. In this case, correct length positioning avoided covering the ostium of the SMA. Only a nonsignificant stenosis < 5% was noted on follow-up CT, with no hemodynamic disturbance and, most importantly, above all, no signs of mesenteric ischemia during the entire 8-month follow-up. Use of three-dimensional imaging can also be helpful since it allows us to see the aneurysm and its relation to the aortic wall and other arterial trunks.

The major limitation of this report is the lack of long-term data on the evolution of the aneurysm, despite encouraging results at 8-month follow-up. However, in the event of inadequate treatment, aortic stenting or surgery can always be performed at a later stage.

In conclusion, embolization of a wide-necked aneurysm of the renal artery ostium that presented as a saccular aneurysm of the abdominal aorta using an AVP 2 and a steerable sheath was successful, with no complications, and obviated the need for a surgical repair or an aortic stent graft.

## Data Availability

All data generated or analyzed during this study are included in this published article.

## Abbreviations

AVP: Amplatzer vascular plug
CBCT: Cone-beam computed tomography
CT: Computed tomography
RAA: Renal artery aneurysm
SMA: Superior mesenteric artery
